# Mitochondrial Polyadenylation Is a One-Step Process Required for mRNA Integrity and tRNA Maturation

**DOI:** 10.1371/journal.pgen.1006028

**Published:** 2016-05-13

**Authors:** Ana Bratic, Paula Clemente, Javier Calvo-Garrido, Camilla Maffezzini, Andrea Felser, Rolf Wibom, Anna Wedell, Christoph Freyer, Anna Wredenberg

**Affiliations:** 1 Department of Mitochondrial Biology, Max Planck Institute for Biology of Ageing, Cologne, Germany; 2 Department of Medical Biochemistry and Biophysics, Karolinska Institutet, Stockholm, Sweden; 3 Department of Molecular Medicine and Surgery, Karolinska Institutet, Stockholm, Sweden; 4 Centre for Inherited Metabolic Diseases, Karolinska University Hospital, Stockholm, Sweden; The University of Western Australia, AUSTRALIA

## Abstract

Polyadenylation has well characterised roles in RNA turnover and translation in a variety of biological systems. While polyadenylation on mitochondrial transcripts has been suggested to be a two-step process required to complete translational stop codons, its involvement in mitochondrial RNA turnover is less well understood. We studied knockdown and knockout models of the mitochondrial poly(A) polymerase (MTPAP) in *Drosophila melanogaster* and demonstrate that polyadenylation of mitochondrial mRNAs is exclusively performed by MTPAP. Further, our results show that mitochondrial polyadenylation does not regulate mRNA stability but protects the 3' terminal integrity, and that despite a lack of functioning 3' ends, these trimmed transcripts are translated, suggesting that polyadenylation is not required for mitochondrial translation. Additionally, loss of MTPAP leads to reduced steady-state levels and disturbed maturation of tRNA^Cys^, indicating that polyadenylation in mitochondria might be important for the stability and maturation of specific tRNAs.

## Author Summary

The polyadenylation of cellular RNAs is a well-studied signal for gene expression, with a defined function in either RNA turnover or translation, in the majority of systems. In mammalian mitochondria the role of polyadenylation is less clear, and can to date only be attributed to completing the translational stop signal on several mitochondrial transcripts. Previous work though demonstrated that mitochondrial polyadenylation requires a certain length and shortening of the poly(A) tail signal has detrimental effects on mitochondrial function. In this study we deleted the mitochondrial polymerase responsible for polyadenylation in the fly, *Drosophila melanogaster*, and demonstrate that the mitochondrial poly(A) tail is essential for preserving the 3’ ends of mitochondrial transcripts, with no other enzyme capable of completing stop signals. Our study also shows that polyadenylation does not regulate transcript stability nor is it required for translation, but might be involved in the maturation of certain mitochondrial tRNAs. We therefore conclude that besides completing translational stop signals, mitochondrial polyadenylation protects the 3’ termini from degradation.

## Introduction

The mitochondrial genome (mtDNA) is well conserved among metazoans, encoding a core set of 11 mRNAs, 22 tRNAs and 2 rRNAs in all species. In general, these genomes are very compact circular molecules, present in almost all metazoan cells as multiple copies within the mitochondrial matrix. Genes are intronless and a single major noncoding region contains regulatory elements, such as promoters and an origin of replication. Transcription from this region results in large polycistronic transcripts, often spanning the majority of the genome and requiring the recruitment of specific processing machineries to release the individual transcripts. Mt-tRNAs are disbursed throughout the genome and are proposed to form the structural basis for the successive cleavage of the 5' and 3' ends by RNaseP and RNaseZ complexes, respectively, at tRNA-mRNA junctions [1–4]. All mitochondrial RNAs require additional post-transcriptional modifications, catalysed by highly specialised enzymes [5,6]. The extent of this transcript maturation varies among species, but the majority of transcripts undergo polyadenylation, with several mRNAs requiring the addition of adenines to complete a translational stop codon. The mammalian ribosomal subunits seem to contain no to little poly(A) tail, while both subunits in flies are polyadenylated. Similarly, both human [7] and murine [8] *MTND6* contain no poly(A) tail, while MTND6 of *Drosophila melanogaster* (*Dm*) is polyadenylated [9,10].

Mitochondrial polyadenylation is catalysed by a designated polyadenylic acid RNA polymerase (MTPAP) [11], transferring on average 40–50 adenines to almost all mitochondrial transcripts [2]. The crystal structure revealed that MTPAP functions as a homodimer without the need for additional protein co-factors [12,13]. However, unlike cytosolic or bacterial polyadenylation signals [14,15], the role of the mitochondrial poly(A) tails is less clear. Silencing of human *MTPAP* in cell culture failed to reveal a uniform function of polyadenylation for transcript stability, but rather led to the suggestion of a transcript-specific response. Loss of poyadenylation appears to stabilise the transcripts for the complex I subunits MTND1 and MTND2, while destabilising mitochondrially encoded complex IV transcripts. On the other hand, other transcripts do not seem to be affected at all by decreased polyadenylation [11,16]. Similar results were obtained in fibroblast cells from a patient with spastic ataxia and optic atrophy due to a pathogenic N478D substitution in MTPAP [17]. This mutation disrupts the fingers domain of MTPAP, severely affecting adenylase function and leading to increased and decreased mRNA steady state levels [18]. None of the above studies though completely abolished MTPAP function, resulting in the presence of short oligoadenylase signals. The failure to detect mitochondrial transcripts devoid of any adenine addition lead to the suggestion that mitochondrial polyadenylation is a two-step process, requiring an initial oligoadenylation by a yet unknown oligoadenylase [5–7,11,19,20]. On the other hand, low processivity, or a balanced activity of adenylation and deadenylation, as reported in Arabidopsis [21], could also explain these results. Nevertheless, in all the above models, mt-mRNAs retained their stop codons, but oligoadenylation was not sufficient for adequate translation in mitochondria [18], raising the question why oligoadenylation is not sufficient for mitochondrial translation. In contrast, a mild increase in poly(A) tail length was observed in cell lines upon silencing or overexpression of mutant components of the proposed mitochondrial degradosome, although no functional consequences were associated [16,20,22].

We previously reported that a number of different steps in the mitochondrial gene expression process can influence the polyadenylation of mt-RNA, with varying consequences on mitochondrial translation [8,10,23]. Silencing the mitochondrial helicase SUV3 in *D*. *melanogaster* led to the accumulation of processing intermediates of mitochondrial primary transcripts, increased mt-mRNA steady-state levels, disrupted translation, and was accompanied by reduced poly(A) tail length [23]. A similar effect on polyadenylation was also observed in knockout and knockdown models of the mammalian leucine-rich pentatricopeptide repeat containing (LRPPRC) protein [8] or its fly homolog bicoid stability factor (BSF) [10], but resulted in increased *de novo* translation. Mutations in LRPPRC have been associated with a severe neurological autosomal recessive neurodegenerative disorder, Leigh syndrome French-Canadian variant [24], and thus it is plausible that reduced polyadenylation plays a part in disease aetiology. LRPPRC forms a complex with the SRA stem loop interacting RNA-binding protein, SLIRP [8,18,25], but unlike LRPPRC/BSF, SLIRP is dispensable for polyadenylation and mt-RNA stability [26]. Thus, it remains unclear why mitochondria require a poly(A) signal of certain length and in what way polyadenylation is involved in mitochondrial translation.

To address these questions we deleted MTPAP in *D*. *melanogaster*. We demonstrate that mitochondrial poly(A) tail formation is exclusively performed by MTPAP and its disruption leads to trimming of the 3' termini and the addition of short heterooligomers to mt-mRNAs. Additionally, we show that, unlike its function in the cytosol, polyadenylation in mitochondria is not required for translation, but the trimmed transcripts fail to encode proteins that result in functional OXPHOS complexes. Finally, our data suggest that mitochondrial polyadenylation does not regulate transcript stability, but rather protects mRNA integrity and might be required for the maturation of mt-tRNA^Cys^.

## Results

### *CG11418* encodes for the mitochondrial poly(A) polymerase and is essential for fly survival

**Fig 1:**
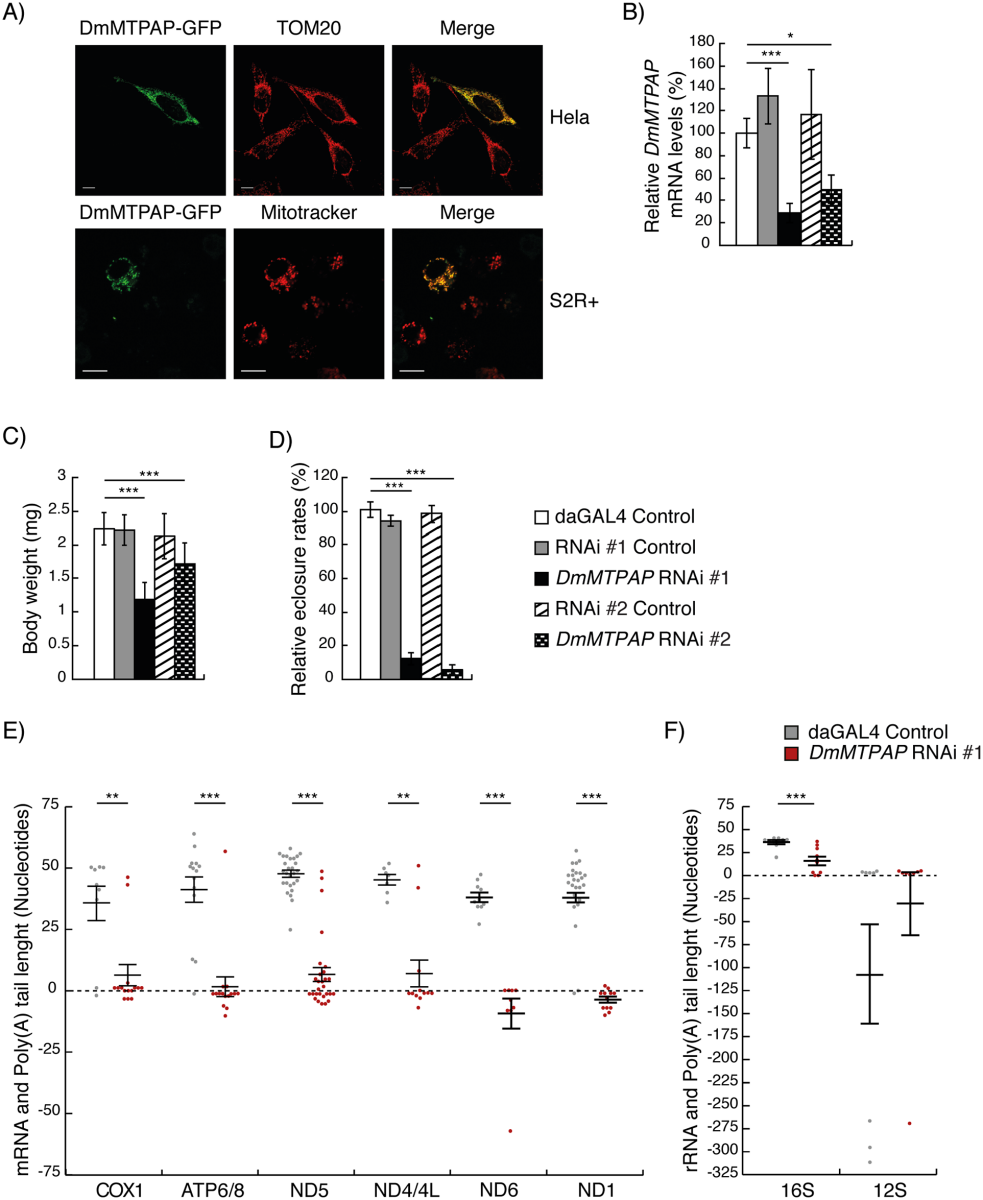
*CG11418* is an essential mitochondrial poly(A) polymerase. (**A**) Immunocytochemistry of HeLa cells (top panel) and Schneider 2R+ (S2R+, bottom panel) expressing a GFP-tagged CG11418 fusion protein (DmMTPAP-GFP). The mitochondrial network was visualised with TOM20 antibodies in HeLa cells or Mitotracker Red in S2R+ cells, respectively. Scale bars represent 20 μm (top panel) and 5 μm (bottom panel). (**B**) Ubiquitous silencing of *CG11418* resulted in significant reduction of *CG11418* transcript levels in both *DmMTPAP* KD lines (black and checked bars) in contrast to the control lines (white, grey and striped bars). Transcript levels were normalised to cytosolic ribosomal protein 49 (RP49) mRNA levels in 5 days after egg laying (ael) larvae (n = 5). Data are represented as mean ± SD (***P < 0.001, *P < 0.05). (**C**) Body sizes of *DmMTPAP* KD (black and checked bars) were significantly larger than control 6-day-ael larvae (white, grey and striped bars) (n = 20). Data are represented as mean ± SD (***P < 0.001). (**D**) The relative eclosure rates of *DmMTPAP* KD flies (black and checked bars) were significantly lower, when compared to control flies (white, grey and striped bars) (n = 5). Data are represented as mean ± SD (***P < 0.001). Individual mitochondrial (**E**) mRNAs or (**F**) rRNAs from 5 days ael larvae were cloned and sequenced to determine poly(A) tail length of various transcripts in *DmMTPAP* KD (red, *DmMTPAP* RNAi #1) and control (grey; daGAL4 control). Mean poly(A) tail length in control samples varied between 35 and 49 adenines (grey; n = 4–14), while *DmMTPAP* KD samples had mainly reduced poly(A) tail lengths. The annotated 3' termini of the indicated transcripts was set to zero to determine poly(A) tail length. Data are represented as mean ± SEM. (*P < 0.05, **P < 0.01, ***P < 0,001), using a Mann-Whitney test.

We performed a standard BLAST search for the human MTPAP ortholog in *Dm* and identified a single candidate, encoded by *CG11418*, sharing a 30% identity on protein level (S1 Fig). Mitochondrial localisation was predicted *in silico*, using either Mitoprot II (0.87) or Target P (0.84) software and confirmed by transiently expressing a GFP-tagged CG11418 fusion protein in both Schneider and HeLa cells (Fig 1A). To assess whether CG11418 has an important role in mitochondrial polyadenylation, we used the UAS-GAL4 enhancer trap system in two independent RNAi lines to down regulate the expression of *CG11418* in flies. Constitutive expression of GAL4 resulted in significant silencing of *CG11418* (Fig 1B), causing reduced larval body weight (Fig 1C) and death at the ferrate stage or soon after eclosure (Fig 1D), suggesting that CG11418 is essential for fly survival. We determined the poly(A) tail length of circularised mitochondrial transcripts in control and knockdown larvae by cloning and sequencing. As previously observed [10,23], the majority of control transcripts showed polyadenylation with 35–50 adenines, including 16S rRNA, while 12S rRNA showed a much shorter poly(A) tail. In contrast, transcripts from CG11418 knockdown larvae revealed a severe reduction in polyadenylation (Fig 1E), prompting us to conclude that *CG11418* is indeed the fly ortholog of human *MTPAP* and will therefore be called DmMTPAP henceforth.

### No evidence of oligoadenylation of mitochondrial transcripts

As mentioned above circumstantial evidence suggested that mitochondrial polyadenylation might be a two-step process [5–7,11,19,20], and in agreement with this, cloning and sequencing of mitochondrial transcripts from silenced DmMTPAP larvae disclosed a polyadenylation signal compatible with oligoadenylation in *MTND5*, *MTCOX1* (Fig 1E) and 16S rRNA transcripts (Fig 1F). In contrast, the majority of cloned transcripts of *MTATP6/8*, *MTND1*, *MTND4/4L* and *MTND6* had no poly(A) tail signal, or transcripts with trimmed 3' termini (Fig 1E). Surprisingly, polyadenylation of 12S rRNA did not seem to be affected by MTPAP silencing and retained on average four adenines on full-length transcripts (Fig 1F).

**Fig 2:**
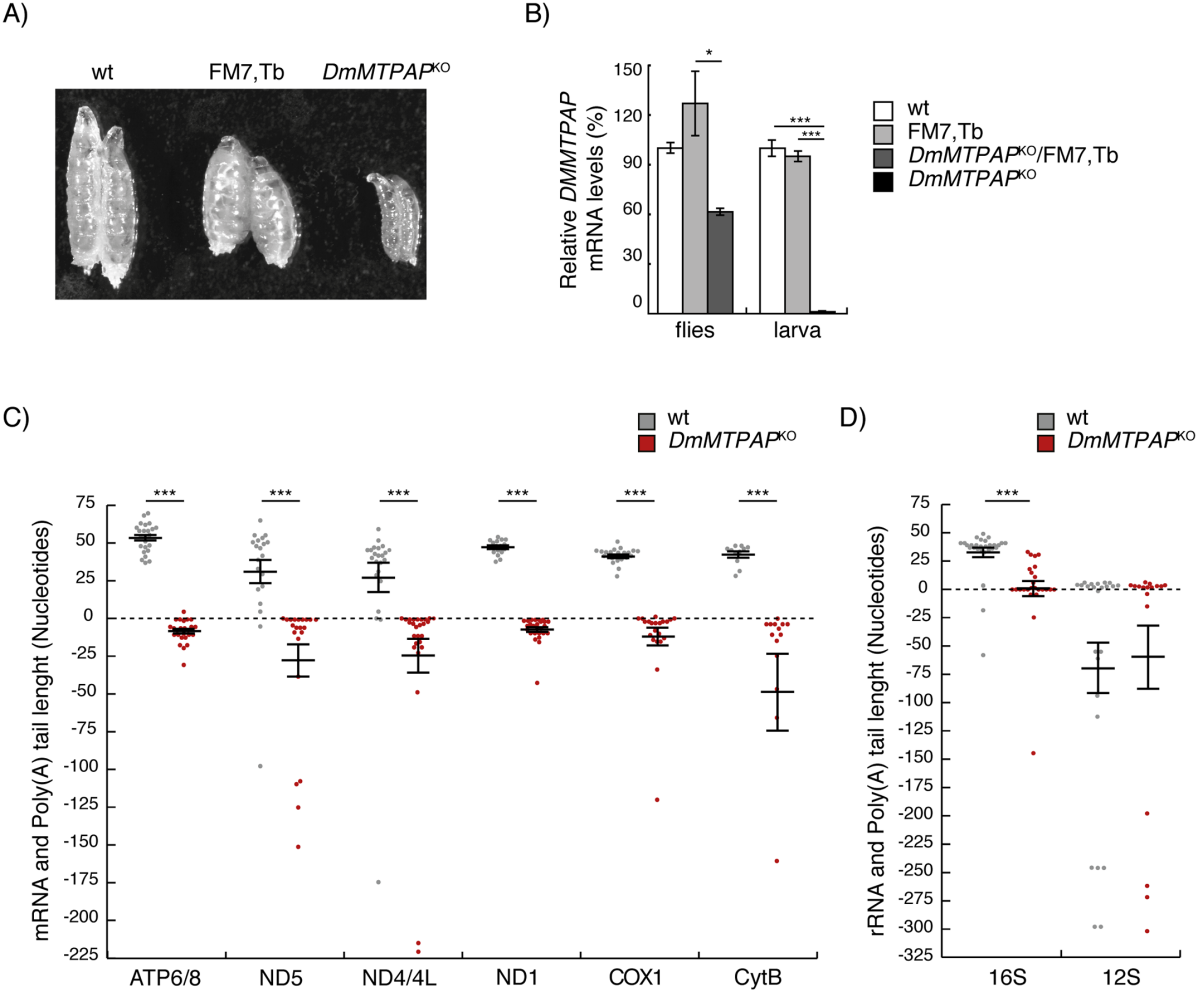
MTPAP is the only mitochondrial adenylase in flies and is required to protect the 3' termini of mRNAs. (**A**) Body size comparison in control (wt and FM7,Tb) and DmMTPAP KO larvae (*DmMTPAP*^KO^) at 4 days ael. (**B**) qRT-PCR analysis of *DmMTPAP* transcript levels in 1 day heterozygous *DmMTPAP*^KO^ flies (*DmMTPAP*^KO^/FM7,Tb) and 4-day-old hemyzygous *DmMTPAP*^KO^ larvae (*DmMTPAP*^KO^) and their corresponding age-matched controls (wt, FM7,Tb). Histone 2B transcript was used as endogenous control. Data is represented as mean ± SEM (*P < 0.05, ***P < 0.001, n = 5). (**C**) mRNA and poly(A) tail length in individually sequenced clones after transcript circularisation (*MTATP6/8*, *MTND4/4L*, *MTND1* and *MTND5*) or 3' RACE (*MTCOX1* and *MTCYTB*) in *DmMTPAP*^KO^ (red, n = 14–26) and control larvae (grey, n = 11–25) at 4 days ael. The annotated 3' termini of the indicated transcripts was set to zero to determine poly(A) tail length. (**D**) rRNA and poly(A) tail length in individually sequenced clones after transcript circularisation in *DmMTPAP*^KO^ (red, n = 17–25) and control larvae (grey, n = 24–29) at 4 days ael. Data are represented as mean ± SEM. (***P < 0,001), using a Mann-Whitney test.

Gene silencing thus left a certain amount of ambiguity for the polyadenylation process of mitochondrial transcripts, and we therefore generated functional *DmMTPAP* knockout (*DmMTPAP*^KO^) flies by homologous recombination, without disturbing the expression of flanking genes or a gene situated within the *CG11418* locus (for cloning strategy and targeting see materials and methods and S2A–S2F Fig). *CG11418* is X-linked and heterozygous *DmMTPAP*^KO^ female flies were viable and fertile, while male *DmMTPAP*^KO^ larva died at the 3rd instar larval stage (Fig 2A). *DmMTPAP* expression levels confirmed that heterozygous female flies retained 50% mtPAP transcript levels, while *DmMTPAP*^KO^ larvae had negligible levels (Fig 2B). Cloning and sequencing of the 3' termini of circularised mitochondrial transcripts from *DmMTPAP*^KO^ larvae failed to show any sign of oligoadenylation, with the exception of one *MTATP6/8* clone and one *MTCOX1* clone that contain 4 or 1 adenine extensions, respectively (Fig 2C). Further, the majority of clones were shortened at the 3' termini by 1–20 nt (Fig 2C). The only exceptions to this were a single *MTND5* clone, three *MTND4/4L* clones, three *MTCOX1* clones and one *MTCYTB* clone, which showed no adenine additions but retained their annotated full-length reading frame (Fig 2C). Some 16S ribosomal transcript subunits still retained full-length poly(A) tail signal, although the majority had reduced or no poly(A) tail signal but no increased amount of 3' trimming. In agreement with the results obtained by *DmMTPAP* silencing, 12S rRNA transcripts were adenylated comparable to controls (Fig 2D). Surprisingly, approximately 30% of the sequenced clones, including the full-length clones from *MTCOX1* and *MTCYTB*, had short oligonucleotide additions, consisting predominantly of cytosine and adenosine nucleotides (S1 Table). To confirm that these additions were indeed 3' extensions, the 3' ends of *MTCOX1* and *MTCYTB* were analysed through a rapid amplification of cDNA ends (RACE), confirming both 3' shortening and unconventional extension of mitochondrial mRNAs upon loss of DmMTPAP (Fig 2C and 2D, S1 Table). Thus, our results demonstrate that MTPAP is the sole adenylase in mitochondria, and loss of DmMTPAP leads to mitochondrial transcripts with compromised integrity.

### The presence of incomplete mitochondrial mRNAs does not promote a general transcript degradation

**Fig 3:**
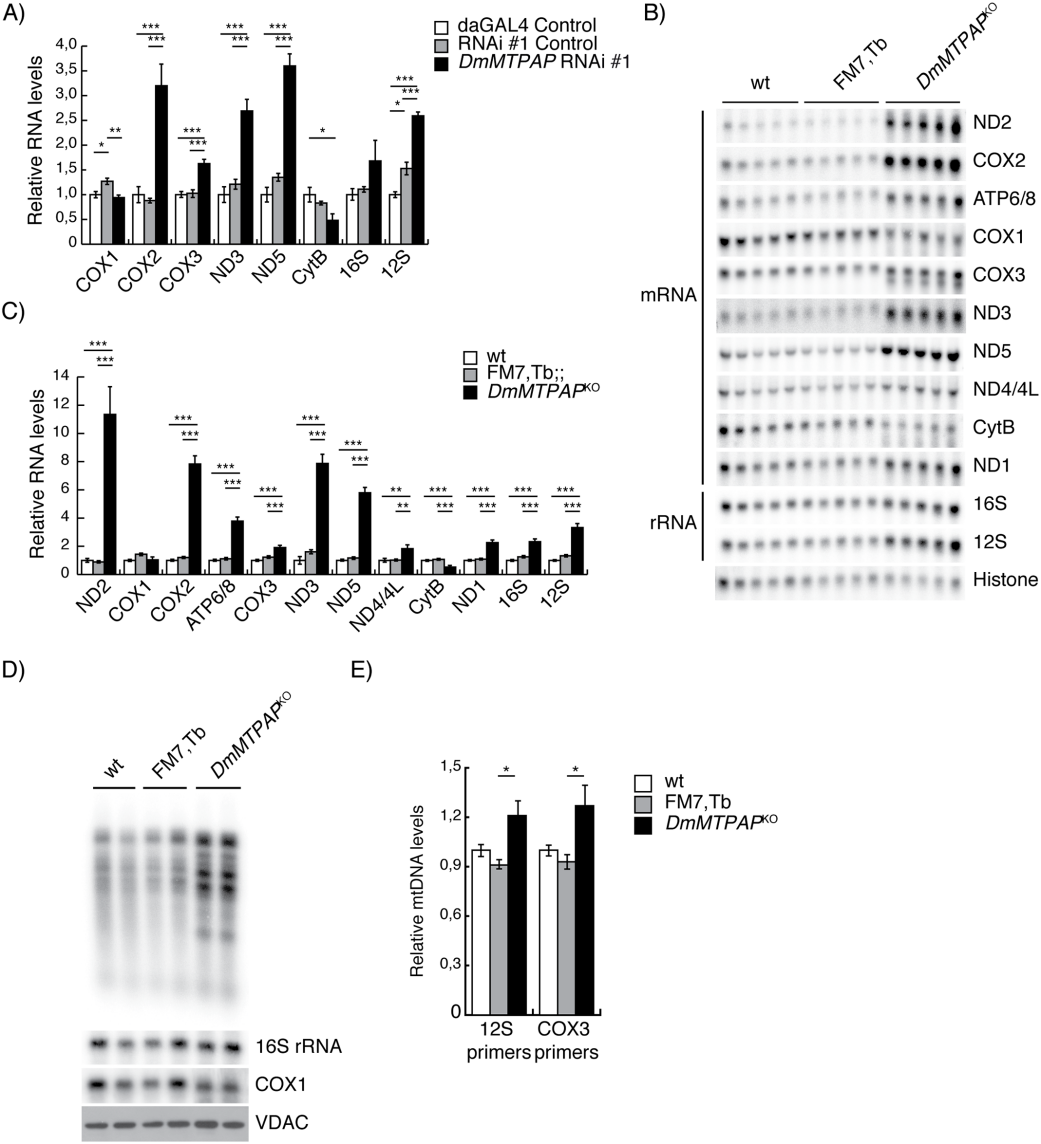
Impaired 3' termini do not affect the stability of most mtDNA-encoded transcripts. (**A**) Relative steady-state level of mitochondrial transcripts were determined by Northern Blot in 5 day ael control (white and grey bars) and *DmMTPAP* KD larvae (black bar) larvae (n = 5). Expression levels were quantified using a Typhoon phosphorimager and normalised to histone 2B mRNA. All data are represented as mean ± SEM. (*P < 0.05, **P < 0.01, ***P < 0,001). (**B**) Northern blot analysis and (**C**) quantification of steady-state levels of mitochondrial transcripts in control (wt and FM7,Tb) and *DmMTPAP* KO larvae (*DmMTPAP*^KO^) at 4 days ael. Histone 2B transcript was used as loading control. (**D**) *De novo* mitochondrial transcription in isolated mitochondria of control and *DmMTPAP* KO larvae at 4 days ael in the presence of radioactively labelled [^32^P]-UTP. Loading of the gels and absence of RNA degradation was confirmed by Northern blotting against COX1 and 16S RNAs. Western blotting of VDAC in the input samples was used as a loading control. (**E**) qPCR of mtDNA steady-state levels *DmMTPAP* KO and control larvae at 4 days ael. Primers against the cytosolic ribosomal protein 49 (RP49) were used to normalise to nuclear DNA content of the samples.

Silencing of human *MTPAP* has previously been suggested to both increase and decrease mitochondrial transcript steady-state levels, while retaining a short oligoadenylation signal [11,16]. Concurrently, the knockdown (Fig 3A) and knockout (Fig 3B and 3C) of *DmMTPAP* in larvae led to an increase of mitochondrial mRNA steady-state levels, with the exception of *MTCOX1* and *MTCYTB*, which showed either unchanged or decreased steady-state levels (Fig 3A–3C). Northern blot analysis also revealed additional shortened transcripts of *MTCOX1*, *MTCYTB*, *MTCOX3* and *MTND4/4L*, suggesting that in the absence of a poly(A) tail degradation intermediates of these transcripts accumulate (S3A Fig). Interestingly, these mRNAs also had comparatively mild changes in steady-state levels in *DmMTPAP*^*KO*^ larvae (Fig 3B and 3C). To confirm the shortened transcripts are indeed the result of mRNA degradation, rather than accumulation of antisense RNAs, we used single-stranded probes against *MTCYTB* confirming the accumulation of shortened transcripts (S3A Fig). The general increase in mRNA steady-state levels in *DmMTPAP*^*KO*^ larvae can be a consequence of *de novo* transcription and indeed, we observed a mild increase in newly synthesized transcripts (Fig 3D) and mtDNA steady-state levels (Fig 3E), however not to such an extent that it can explain the increased steady-state levels of mitochondrial transcripts. Thus, despite loss of 3' integrity, mitochondrial mRNAs were not degraded by the mitochondrial degradosome, suggesting that polyadenylation is required for sufficient degradation of some mitochondrial transcripts.

### MTPAP activity is required for the maturation and stability of tRNA^Cys^

**Fig 4:**
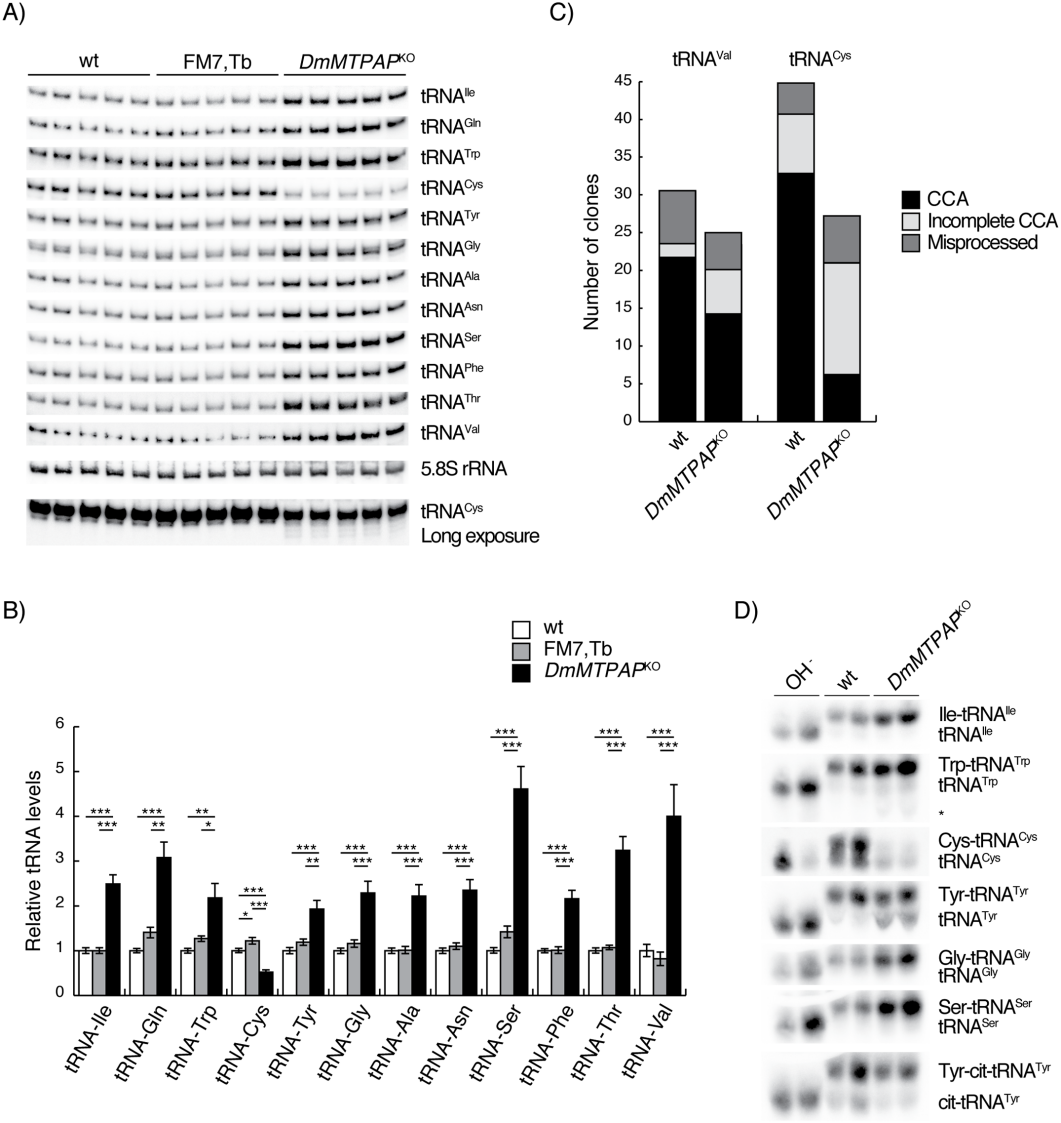
tRNA^Cys^ is not maturing correctly in the absence of MTPAP. (**A**) Neutral PAGE, Northern Blot analysis and (**B**) quantification of mitochondrial tRNA steady-state levels of *DmMTPAP* KO larvae (*DmMTPAP*^KO^) and control (wt and FM7,Tb) larvae at 4 days ael. rRNA 5.8S was used as a loading control. Data is represented as mean ± SEM (*P < 0.05, **P < 0.01, ***P < 0,001, n = 5). (**C**) 3' end sequencing of individual clones of tRNA^Val^ and tRNA^Cys^ in 3' RACE experiments of control (wt) and *DmMTPAP*^KO^ RNA samples at 4 days ael. tRNAs were grouped according to the presence of the complete CCA tail (CCA, black), incomplete CCA (CC- or C-, pale grey) or incorrect tail (detailed in S3 Table, dark grey). (**D**) Aminoacylation status of mitochondrial tRNAs was analysed in acid-urea PAGE followed by Northern blotting in 4-day-old control (wt) and *DmMTPAP*^KO^ larvae. Basic conditions (OH-) deaminoacylate all of the analysed tRNAs. Cytosolic tRNA^Tyr^ (cit-tRNA^Tyr^) was used as a control of tRNA aminoacylation in each sample.

Transfer RNA steady-state levels are sometimes seen as a proxy for *de novo* transcription, and in agreement with *in organello* experiments, mitochondrial tRNA levels were all increased in *DmMTPAP*^*KO*^ larvae. The only exception was tRNA^Cys^, which showed a marked reduction and an accumulation of shortened RNAs (Fig 4A and 4B). A similar trend was observed in RNAi knockdown larvae (S3B Fig), although no increase in *de novo* transcription (S3C Fig) or mtDNA levels (S3D Fig) was observed. To address the difference in stability of tRNA^Cys^, we cloned and sequenced tRNA^Cys^ transcripts, showing that the majority of transcripts lacked the usual CCA addition in *DmMTPAP*^*KO*^ larvae (Fig 4C, S2 Table). This maturation defect was confirmed by a lack of aminoacylation of tRNA^Cys^, with tRNA^Tyr^ being the only other tRNA affected (Fig 4D).

### Intact mRNAs are not required for mitochondrial translation

**Fig 5:**
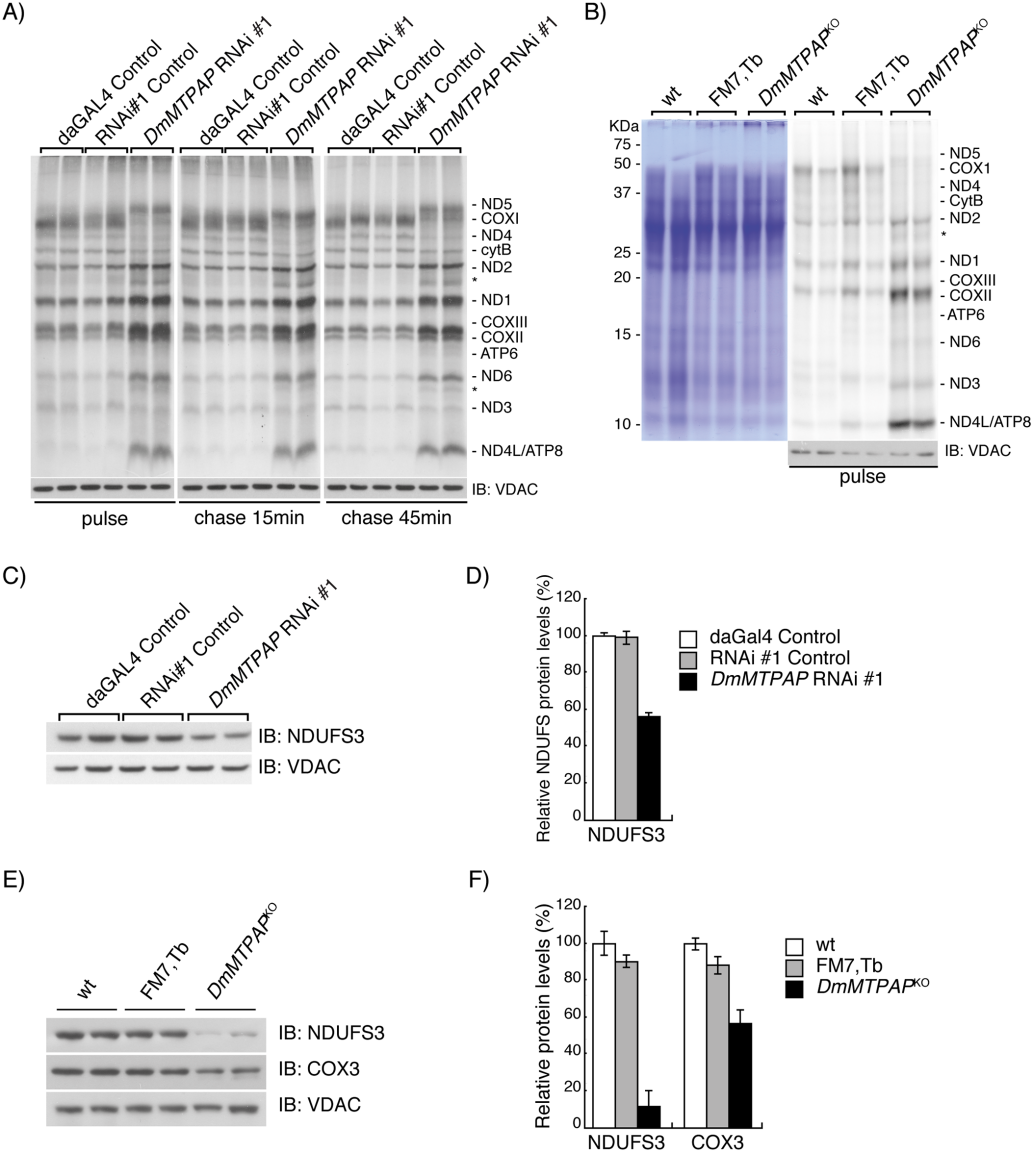
Polyadenylation is not required for mitochondrial translation. (**A**) *In organello* labelling of mitochondrial translation products on isolated mitochondria from *DmMTPAP* KD (*DmMTPAP* RNAi #1) and control (w;;daGAL4/+ and w;UAS-mtPAPRNAi#1/+) 5 days ael larvae. Labelling was performed for 60min (pulse), followed by a 15 or 45 min chase with cold methionine. Loading was normalised to VDAC levels. (**B**) *In organello* labelling of mitochondrial translation products on isolated mitochondria from *DmMTPAP* KO (*DmMTPAP*^KO^) and control (wt and FM7,Tb) 4-day-old larvae. Coomassie staining of the gels and VDAC Western blotting of the input samples were performed to ensure equal loading of the samples. Western blot analysis (**C**) and quantification (**D**) of nuclear-encoded subunit of Complex I (NDUFS3) in isolated mitochondria from control (daGAL4 control, RNAi #1 control and RNAi#2 control) and *DmMTPAP* KD (*DmMTPAP* RNAi #1 and *DmMTPAP* RNAi #2) 5-day-old larvae. VDAC was used as a loading control. Western blot analysis (**E**) and quantification (**F**) of the steady-state levels of a nuclear-encoded subunit of Complex I (NDUFS3) and an mtDNA-encoded subunit of complex IV (COX3) in mitochondria of control (wt and FM7,Tb) and *DmMTPAP*^KO^ 4-day-old larvae. VDAC was used as a loading control. Data are represented as mean ± SD.

Several mitochondrial transcripts require polyadenylation to complete a functional stop codon, but even oligoadenylation was insufficient for normal translation in human mitochondria from a patient with mutant MTPAP, suggesting a requirement of polyadenylation for translation [18]. We were therefore interested to investigate the effects on translation in the absence of full-length mRNAs. To our surprise, we observed an aberrant pattern with increased levels of *de novo* translation for the majority of peptides, both in KD (Fig 5A and S4 Fig) and KO (Fig 5B) mitochondria. We also observed some truncated peptides, suggesting aberrant translation for some peptides (Fig 5A and 5B, asterisk). Interestingly, translation seemed to correlate with transcript steady-state levels, with decreased MTCOX1/MTND4/MTCYTB polypeptides. Despite the increased *de novo* translation, we observed decreased steady-state levels of the nuclear-encoded complex I subunit NDUFS3, both in RNAi (Fig 5C and 5D) and *DmMTPAP*^*KO*^ samples (Fig 5E and 5F), as well as of the mtDNA-encoded complex IV subunit MTCOX3 (Fig 5E and 5F), suggesting impaired complex assembly.

### Loss of mitochondrial polyadenylation leads to a severe mitochondrial dysfunction

**Fig 6:**
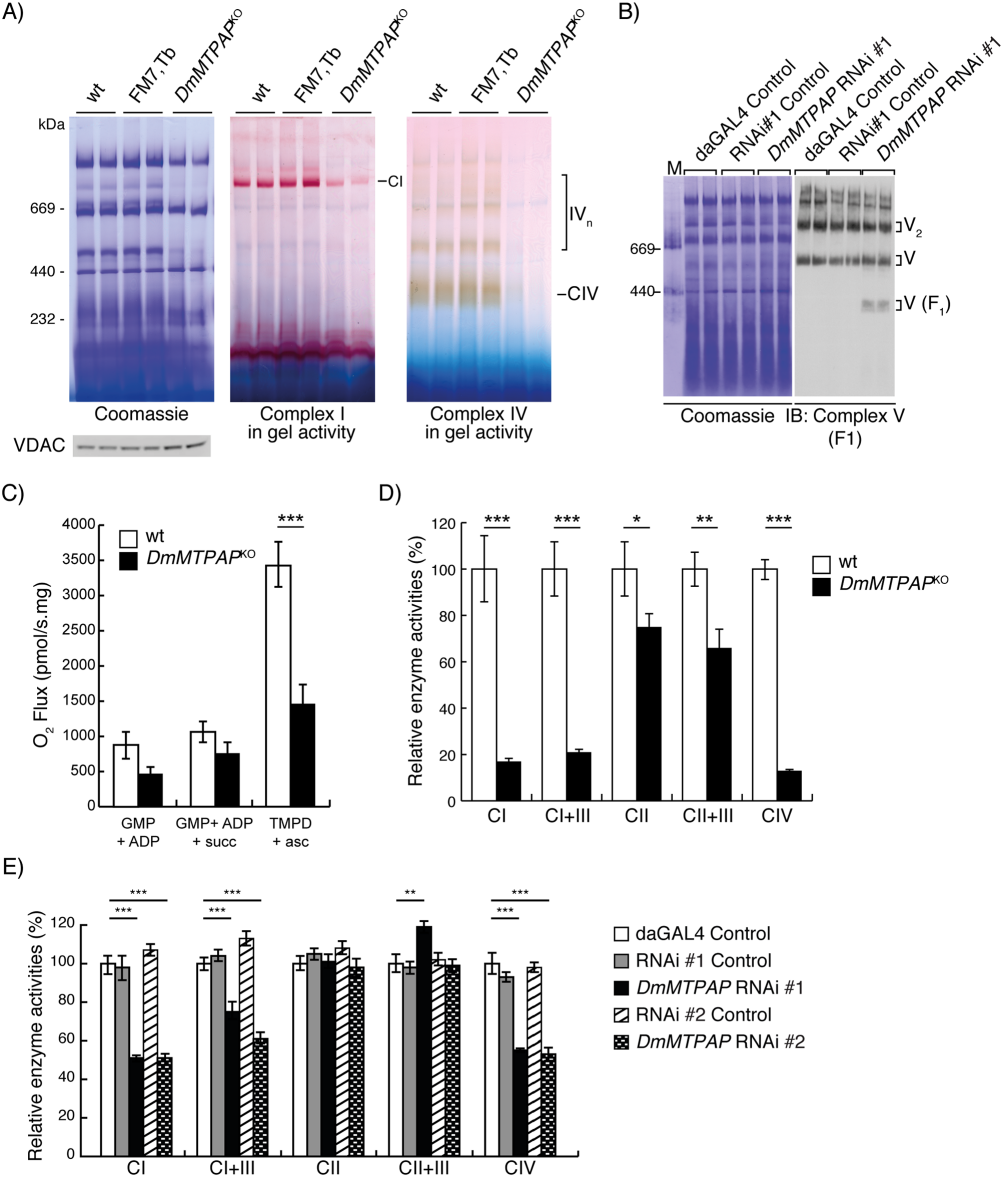
Mitochondrial respiration is affected due to incomplete OXPHOS assembly. (**A**) BN-PAGE and in gel staining of Complex I and Complex IV activities in mitochondrial protein extracts from control (wt and FM7,Tb) and DmMTPAP KO (*DmMTPAP*^KO^) 4-day-old larvae. Coomassie staining of the gel and VDAC western blot of the input samples was performed to ensure equal loading of the gel. (**B**) Complex V assembly was assessed in *DmMTPAP* KD (*DmMTPAP* RNAi #1) 5-day-old larvae by BN-PAGE, followed by Western blot analysis against the F1 subunit of Complex V. Coomassie staining was used to ensure equal loading. (**C**) Oxygen consumption rates in permeabilised 4-day-old control (wt) and *DmMTPAP*^KO^ larvae, using glutamate, malate and pyruvate (GMP + ADP), succinate (GMP + ADP + succ) or TMPD and ascorbate (TMP + asc) as electron donors. Data are normalized to the protein content in each sample and are represented as mean ± SEM (***P < 0,001, n = 8). (**D**) Relative enzyme activities of respiratory chain complexes in 4-day-old control (wt) and *DmMTPAP*^KO^ larvae. Data are represented as mean ± SD (*P<0.05, **P < 0.01, ***P < 0,001, n = 3). (**E**) Relative enzyme activities of respiratory chain complexes (Complex I-IV) from control (white, grey and striped bars) and *DmMTPAP* KD (checked and black bars) 5-day-old larvae. Data is represented as mean ± SEM (**P < 0.01, ***P < 0,001, n = 5).

To assess OXPHOS complex formation, we performed blue-native gel electrophoresis (BN-PAGE) experiments, revealing reduced complex assembly and decreased complex I and IV in-gel activities, both in RNAi (S5A and S5B Fig) and *DmMTPAP*^*KO*^ (Fig 6A) mitochondria. Additionally, Western blot analysis also exposed a complex V assembly defect (Fig 6B and S5C Fig). Finally, we performed mitochondrial oxygen consumption measurements, demonstrating that the loss of polyadenylation leads to a combined complex I and IV defect (Fig 6C), confirmed by decreased isolated respiratory chain enzyme activities in both *DmMTPAP*^*KO*^ and RNAi larvae (Fig 6D and 6E).

## Discussion

Despite extensive efforts, the role of mitochondrial polyadenylation remains elusive, with both stabilising and destabilising roles assigned to various mitochondrial transcripts [11,16–18]. How these differential roles are supposedly regulated or executed is unclear, and therefore the only known function of mitochondrial polyadenylation is that it serves to complete the stop codon of several mitochondrial-encoded transcripts. To gain insights into the role of mitochondrial polyadenylation we identified the *Drosophila melanogaster* homolog *CG11418* as the fly ortholog to human MTPAP, and show that polyadenylation is essential for mitochondrial function and fly development.

Silencing of MTPAP leads to reduced poly(A) tail length in human cell lines [11,16], while cells from a patient with a mutation in MTPAP also severely affected polyadenylation [17,18]. In these experiments, all transcripts retained short adenine tails, leading to the proposal that mitochondrial polyadenylation is a two-step process, requiring an oligoadenylase to initiate polyadenylation. However, although silencing of *DmMTPAP* also retained very short polyadenylation signals for some transcripts in flies, deletion of *DmMTPAP* resulted in a complete loss of polyadenylation signal in all mitochondrial mRNAs investigated. The only transcripts retaining some degree of polyadenylation in KO flies were the two ribosomal transcripts. We interpret this that maternal ribosomal transcripts are protected in the monosome from degradation and therefore retained their tail. This is supported by the observation that 16S rRNA transcripts showed one population of transcripts with poly(A) tail—albeit shortened—and one population with no tail at all. On the other hand, the presence of poly(A) tails on ribosomal RNAs could also be explained by the presence of an rRNA-specific adenylase. This interpretation is supported by the fact that 12S rRNA showed no change in poly(A) tail length, with no transcripts completely lacking polyadenylation. Interestingly, 12S rRNA has naturally a short poly(A) tail favouring the idea of a 12S rRNA-specific oligoadenylase. Both of these interpretations are not exclusive, but our results reveal that no oligoadenylase is capable of compensating for the loss of MTPAP on mRNAs.

Previous reports from human cell lines and our observations made in flies demonstrate that silencing MTPAP can result in short poly(A) tails. In the absence of an oligoadenylase, it is intriguing that reduced MTPAP levels do not result in either non-polyadenylated transcripts, or full-length poly(A) tails, but rather in shortened tails. MTPAP therefore might have low processivity *in vivo* or requires a stabilising factor, similar to polyadenylation in the nucleus, where the polyadenylate-binding nuclear protein, PABPN1, binds to the newly synthesised poly(A) tail to increase PAP affinity to RNA [27]. However, no such poly(A) tail binding protein has been identified in mitochondria, and targeting the cytosolic PABPC1 to mitochondria not only inhibited mitochondrial translation and OXPHOS function but also reduced poly(A) tail length [28]. Polyadenylation is suggested to be regulated by the activities of MTPAP and the 2'-phosphodiesterase, PDE12, proposed to deadenylate mitochondrial mRNAs [29]. Disruption of MTPAP might disturb this balance, and the overexpression of PDE12 in human cell lines resulted in reduced poly(A) tail length of some transcripts, although the majority of transcripts were unaffected, suggesting that additional factors might be required. A similar mechanism has been seen in *Arabidopsis*, where a balance between the mitochondrial poly(A)-specific deadenylase, AHG2, and the polyadenylase, AGS1, seem to regulate RNA steady-state levels by modulating the poly(A) signal of mitochondrial mRNAs [21]. Interestingly, PDE12 overexpression also resulted in trimmed 3' termini, suggesting that PDE12 might be able to degrade into the 3' ends passed the poly(A) tail signal [29]. We also observed 3' trimming after deleting *DmMTPAP* in all mRNAs investigated, suggesting that polyadenylation protects the 3' end of mitochondrial RNAs from degradation.

Polyadenylation has previously been proposed to either stabilise or destabilise mitochondrial transcripts, with a stabilising effect on transcripts that require polyadenylation for stop codon formation. Deletion of *DmMTPAP* did not confirm this function, with the lowest steady-state levels observed in *MTCOX1* and *MTCYTB* transcripts, which do encode a functional stop codon in mtDNA, while highest steady-state levels were observed in transcripts both requiring polyadenylation for stop codon formation (*MTND2*, *MTCOX2*, *MTND5*) or already encoding a stop codon (*MTND3*). Additionally, we observed no agreement with previous results of polyadenylation and its effects on transcript stability, suggesting that the poly(A) tail does not differentially regulate mitochondrial transcript stability.

Polyadenylation in bacteria promotes degradation via a short poly(A) tail of ~30nt that resolves the 3' stem loop structure of bacterial mRNAs and a similar function is observed in plant mitochondria [30]. Increased steady-state levels and trimmed 3’ ends of the majority of messenger transcripts in the absence of MTPAP favours the hypothesis that the metazoan poly(A) tail has a similar function. In fact, a previous report suggested that a combination of PNPase and/or MTPAP activity adds short homo- or heterooligomers to mRNAs during the degradation process of mitochondrial transcripts [7]. Failure to add such short tails would potentially allow for certain mitochondrial transcripts to adopt a structural confirmation at the 3’ termini that prevents their degradation. Our results are compatible with such a model, as failure to polyadenylate resulted in increased steady-state levels of the majority of mitochondrial transcripts and transcripts that were truncated by maximal 20nt. An involvement of a structural configuration in the degradation of mitochondrial transcripts is supported by our observation that in the absence of polyadenylation many transcripts did contain an oligomer extension, but unlike the extensions proposed to occur during degradation [7], the extensions observed here carried the signature of the tRNA nucleotidyl transferase (TRNT1), responsible for the CCA-addition during tRNA maturation [31]. Interestingly, the CCA additions by TRNT1 prevent re-processing by RNaseZ [32] and thus might be a protective mechanism. However, our results are also compatible with the presence of factor(s) regulating transcript stability upon polyadenylation. For instance, the Fas-activated serine/threonine kinase, FASTK, has recently been suggested to interact with the 3' termini of MTND6 in human cells, regulating transcript stability [33], while other members of the FASTK family have also been reported to affect transcript steady-state levels of some mt-RNAs [34]. Human MTND6 is not polyadenylated and whether polyadenylation was required for other factors is not known.

In human mtDNA, tRNA^Tyr^ and tRNA^Cys^ overlap by a single base pair and it has recently been proposed that MTPAP activity is required to correctly resolve this overlap at the 3' end of tRNA^Tyr^ [35]. In the fly these two tRNAs do not overlap, but we still observed maturation defects for both tRNAs. tRNA^Cys^ was also the only tRNA investigated with reduced steady-state levels and also presented with incorrect CCA modifications. The mechanism behind this is not clear, but suggests that MTPAP is required for maturation of this cluster in additional species. Despite low steady-state and aminoacylation levels of tRNA^Cys^ mitochondrial translation was upregulated. This is particularly surprising, when considering that the majority of mRNAs encoded incomplete 3' termini, demonstrating that the poly(A) tail is not required for mitochondrial translation. However, these transcripts do not encode functional peptides, which fail to assemble into complexes. Thus, mitochondria might not possess a nonsense mediated decay pathway to remove incorrect mRNAs, and it will be interesting to identify ribosomal release mechanisms in these flies.

In summary, we demonstrate that polyadenylation of metazoan mitochondrial mRNAs is not dependent on a mitochondrial poly(A) oligoadenylase, and that polyadenylation is not required for the stability of transcripts. We rather suggest that the poly(A) tail protects mRNA integrity preserving the 3' termini from degradation.

## Materials and Methods

### MTPAP co-localisation, cell culture and transfection

*DmMTPAP* cDNA was cloned (for primers see S3 Table) into pEGFP-N3 plasmid (Clontech) to generate a *dmmtpap*-GFP fusion construct, which was subsequently subcloned into pAc5.1/V5-His A plasmid (Life Technologies). Constructs were used to transfect HeLa or Schneider 2R+ cells, respectively. HeLa cells were cultured in high-glucose DMEM (Life Technologies) supplemented with 10% foetal bovine serum at 37°C in a 5% CO_2_ atmosphere. Schneider 2R+ cells were cultured in Schneider’s *Drosophila* Medium (Life Technologies) supplemented with 10% foetal bovine serum at 25°C. For co-localization studies, HeLa cells or Schneider 2R+ cells were transfected using a calcium phosphate transfection kit (Sigma-Aldrich), following the manufacturer’s instructions. 48 hours after transfection HeLa cells were fixed with 4% PFA and decorated with anti-TOM20 antibody (Santa Cruz, sc-11415) Schneider 2R+ cells were stained with 50 nM Mitotracker Red (Life Technologies). Images were obtained in a Nikon Confocal Microscope at the Live Cell Imaging unit, Karolinska Institutet.

### *Drosophila* stocks and generation of a DmMTPAP knock-out line by ends-out homologous recombination

The two non-overlapping UAS-RNAi lines w;UAS-*MTPAP* RNAi#1; (#11418-R1) (NIG-Fly Stock Centre (Japan)) and w;UAS-*MTPAP* RNAi#2; (#31497) (Vienna Drosophila Resource Centre) were used for *in vivo* knockdown studies. In all studies, ubiquitous down-regulation of *CG11418* expression was driven by driver *daughterless*GAL4 (w;;daGAL4) analogous to previous reports [10,23,36]. Experimental samples are labeled as follows throughout the text: daGAL4 Control (w;;daGAL4/+), RNAi #1 Control (w;UAS-*MTPAP* RNAi#1/+;), *DmMTPAP* RNAi #1 (w;UAS-*MTPAP* RNAi#1/+;daGAL4/+), RNAi #2 Control (w;UAS-*MTPAP* RNAi#2/+;) and *DmMTPAP* RNAi #2 (w;UAS-*MTPAP* RNAi#1/+;daGAL4/+).

The DmMTPAP knockout line was generated by ends-out homologous recombination as described previously [36,37]. The *CG11418* locus contains a second gene between exons 2 and 3 of *CG11418*, Tsp2A, and the knockout strategy therefore only targeted exon 1 and the 5' end of exon 2 of *CG11418*, removing an 817 bp DNA region that contains the initiation of transcription, the ATG start codon and up to the first in frame ATG codon in position 342 of *CG11418* mRNA. Approximately 5 Kb upstream and 3 Kb downstream of the *CG11418* locus (BAC clone from BACPAC Resource Centre, Oakland, California, USA) were cloned by ET recombination into the pBluescript II SK+ vector (Stratagene). Both homology arms were sequenced and subsequently subcloned into the pGX-attP vector [37], generating pGX-attP/*DmMTPAP*^KO^. Primer sequences and restriction sites are listed in S3 Table. pGX-attP/*DmMTPAP*^KO^ was injected into *D*. *melanogaster* embryos via P-element-mediated germ line transformation using The BestGene Drosophila Embryo Injection Services (Chino Hills, California, USA). Crosses for ends-out homologous recombination were carried out as described [37]. Homologous recombination events were identified by PCR and Southern Blot analysis. Primers for PCR screening and cloning of the Southern Blot probe are detailed in S3 Table.

All fly stocks were backcrossed for at least 6 generations into the white Dahomey (without Wolbachia) background (w;;). All fly lines were maintained at 25°C and 60% humidity on a 12h:12h light:dark cycle on a standard yeast-sugar-agar medium.

### Hatching rates

To estimate the eclosure rates of adult flies, eggs were collected during a 3-hour time window and transferred to vials (100 eggs/vial) to ensure standard larval density. Eclosure of adult flies was monitored in regular time intervals.

### DNA isolation, qPCR and Southern blot analysis

Genomic DNA was isolated with the DNeasy Blood and Tissue Kit (Qiagen), following manufacturer’s instructions. For Southern Blot mapping of *DmMTPAP*^KO^ larvae, 1 μg of each DNA sample was digested with SalI and precipitated, followed by separation on a 0.8% agarose gel and blotting to Hybond-N+ membranes (GE Healthcare). Membranes were hybridized with [^32^P]-labelled double stranded DNA probes and exposed to PhosphorImager screens. dsDNA probes were labelled with [^32^P]-dCTP (Perkin Elmer) following the PrimeIt II kit protocol (Stratagene). qPCR quantification of mtDNA levels was performed in triplicates on a QuantStudio 6 (Applied Biosystems), using 5 ng of DNA and Platinum SYBR Green qPCR supermix-UDG (Life Technologies). Primers for probes and qPCR are listed in S3 Table.

### RNA isolation and quantitative RT-PCR (qRT-PCR)

Total RNA was isolated using the ToTALLY RNA kit (Ambion) and quantified with a Qubit fluorometer (Life Technologies) unless otherwise stated. Reverse transcription for qRT-PCR analysis was performed using High Capacity cDNA Reverse Transcription Kit (Life Technologies). qRT-PCR was performed on a QuantStudio 6 (Life Technologies) and/or 7900HT (Applied) with Taqman probes (Life Technologies) or Platinum SYBR Green qPCR supermix-UDG (Life Technologies) and gene-specific primers. Primers and Taqman probes used for qRT-PCR are listed in S3 Table.

### RNA circularisation and RT-PCR

RNA circularisation and RT-PCR was performed as previously described [9,10,23]. In brief, mitochondrial RNA extracts were treated with TURBO DNase (Life Technologies) to remove contaminant DNA. 12 ng of mitochondrial RNA were circularised with T4 RNA ligase 1 (New England Biolabs), precipitated and cDNA synthesis was performed, using the GeneAmp RNA PCR kit (Life Technologies) and gene-specific primers. PCR products were cloned into pCRII-TOPO and transformed in One Shot TOP10 *E*. *coli* (Life Technologies) following manufacturer's instructions. The plasmid was subsequently purified and the insert was sequenced using M13 forward and M13 reverse primers. Primer sequences for RT-PCR to amplify the region containing the poly(A) tails have been previously described [10,23].

### 3' RACE of mitochondrial mRNAs and tRNAs

Linker ligation was essentially performed as previously described [38]. In brief, a phosphorylated oligonucleotide linker was ligated to 2.5 μg of a total RNA sample using T4 RNA ligase 1 (New England Biolabs). RNA was precipitated and cDNA synthesis was performed using a primer complementary to the linker sequence (anti-linker) and the GeneAmp RNA PCR kit (Life Technologies). The 3' end of the mitochondrial RNAs was PCR amplified using the anti-linker and gene-specific primers. The PCR products were cloned into pCRII-TOPO and transformed in One Shot TOP10 *E*. *coli* (Life Technologies) following manufacturer's instructions. The plasmids were then purified and the insert was sequenced using M13 forward and M13 reverse primers. Linker, anti-linker and gene-specific primers for 3' RACE experiments are listed in S3 Table.

### Northern blot analysis of mitochondrial RNAs

Steady-state levels of mitochondrial transcripts were determined by Northern blot analysis, using 3 μg of total RNA. RNA samples were separated in 1% MOPS-formaldehyde agarose gels and transferred to Hybond-N+ membranes (GE Healthcare). To analyse mitochondrial tRNA steady-state levels, samples were separated in neutral 10% PAGE and transferred to Hybond-N+ membranes (GE Healthcare). Membranes were hybridised with either randomly [^32^P]-labelled dsDNA probes or *in vitro* transcribed single-stranded RNA probes to detect mRNAs and rRNAs or with strand-specific [^32^P]-end labelled oligonucleotide probes to detect tRNAs. Membranes were exposed to a PhosphorImager screen and the signal was quantified using a Typhoon 7000 FLA and the ImageQuant TL 8.1 software (GE Healthcare). Primers used to generate dsDNA probes and oligonucleotide probes have been previously described [10,23,36].

### Aminoacylation status of mitochondrial tRNAs

RNA was isolated from 4-day ael larvae, using TRIzol Reagent (Life Technologies) and resuspended in 0.3 M NaOAc (pH 5), 1 mM EDTA. 2 μg of RNA were loaded on acidic 6.5% polyacrylamyde, 8 M urea, 0.1 M NaOAc pH 5 gels and run for 48h at 4°C. Gels were transferred to Hybond-N+ membranes (GE Healthcare) and tRNAs were blotted as described above. To deaminoacylate tRNAs, samples were incubated in 0.5 M Tris pH 9 at 70°C for 10 minutes before loading on the gels.

### In organello transcription and translation assays

Mitochondria were isolated from third instar larvae and *in organello* transcription assays were performed as previously described [10,23,36]. In brief, 200 μg of fresh mitochondria were incubated for 45 min in transcription buffer (30 μCi [^32^P]-UTP, 25 mM sucrose, 75 mM sorbitol, 100 mM KCl, 10 mM K_2_HPO_4_, 50 μM EDTA, 5 mM MgCl_2_, 1 mM ADP, 10 mM glutamate, 2.5 mM malate, 10 mM Tris-HCl pH 7.4 and 5% (w/v) BSA), followed by RNA extraction, separation on a 1% MOPS-formaldehyde agarose gel and transferring to Hybond-N+ membranes (GE Healthcare). Mitochondrial *de novo* translation in isolated mitochondria was assayed as previously described [10,23,36], using EXPRESS protein labeling mix easy-tag (Perkin Elmer). Equal amounts of total mitochondrial protein were separated on 17% SDS-PAGE gels, followed by staining with 1 g/L Coomassie Brilliant Blue in a 20% ethanol, 10% acetic acid solution. Gels were then destained, dried and exposed to a PhosphorImager screen to visualise the mitochondrial translation products.

### Western blot analysis

Western blot analyses were performed using whole fly or mitochondrial protein extracts according to the Cell Signaling Technology protocol (CellSignaling). Protein extracts were separated on 4–12% or 12% NUPAGE acrylamide gels (Invitrogren) and after transfer to PVDF membranes (Millipore) decorated with the following antibodies: Complex I-subunit NDUFS3 (Mitoscience MS112, dilution 1:1000), complex IV-subunit COX3 (Mitosciences, MS406, 1:500), complex V (Mitoscience MS504, dilution 1:5000) and VDAC (Mitoscience MSAO3, dilution 1:1000–2000). Protein bands were visualized with ECL western blotting reagents (Bio-Rad).

### Blue native polyacrylamide gel electrophoresis (BN-PAGE) and in-gel activity assays

BN-PAGE and in-gel staining for complex I and IV activities was performed as previously described [10,23,36]. In brief, mitochondria were pelleted and lysed in 1% digitonin, 0.1 mM EDTA, 50 mM NaCl, 10% glycerol, 1 mM PMSF and 20 mM Tris pH 7.4 for 15 minutes on ice. After removing undissolved material by centrifugation, samples were loaded on 4–10% polyacrylamide gradient gels. In-gel complex I activity was determined by incubating the BN-PAGE gels in 2 mM Tris-HCl pH 7.4, 0.1 mg/ml NADH and 2.5 mg/ml iodonitrotetrazolium chloride. In-gel complex IV activity was determined by incubating the BN-PAGE gels in 50 mM phosphate buffer pH 7.4, 0.5 mg/ml 3.3’-diamidobenzidine tetrahydrochloride (DAB), 1 mg/ml cytochrome c, 0.2 M sucrose and 20 μg/ml catalase. Staining was performed at room temperature.

### Biochemical evaluation of respiratory chain function

Oxygen consumption from third-instar larvae was measured at 25°C, using an oxygraph chamber (OROBOROS), as previously described [10,36]. Respiratory chain enzyme activities were determined on isolated mitochondria, as previously described [39].

### Statistical analysis

All data are presented as mean ± standard error of the mean (SEM) or standard deviation (SD) as indicated. A one-way ANOVA with Dunnett's multiple comparison test was used for statistical analysis, except for transcript tail length cloning and sequencing data, where a Mann-Whitney test was used.

## Supporting Information

S1 Table Cloning and sequencing results of the 3' termini of mitochondrial transcripts from *DmMTPAP*^KO^, control (daGal4 control), and RNAi knockdown (mtPAP RNAi #1) larvae. (XLSX)

S2 Table tRNA 3’ RACE in control (wt) and *DmMTPAP*^*KO*^ larvae. (DOCX)

S3 Table List of TaqMan probes and oligonucleotides used. (DOCX)

S1 Fig Alignment of human and *Drosophila melanogaster* mitochondrial poly(A) polymerases. Clustal W alignment of human mitochondrial poly(A) polymerase (bottom, AAH61703) and its *D*. *melanogaster* ortholog (top, AAF45607).
(TIF)

S2 Fig Knockout strategy of DmMTPAP. (A) Schematic diagram of the MTPAP locus, the targeting vector for homologous recombination and the KO locus. (B-D) Homologous targeting confirmation by PCR on control (wt and FM7,Tb), heterozygous (*DmMTPAP*^KO^/FM7,Tb) and hemizygous knockout (*DmMTPAP*^KO^) samples. Primers (see M&Ms) were chosen to either span the deleted region (B and PCR1 in S1A) or the homologous arms of the targeting vector (C, D and PCR 2,3 in S1A). (E) Southern blot of SalI digested genomic DNA probed as shown in S1A. (F) qRT-PCR of expression levels of genes adjacent to the deleted MTPAP locus.
(TIF)

S3 Fig Relative mitochondrial RNA steady-state levels. (A) Northern Blot analysis of mitochondrial mRNAs using dsDNA probes (COX1, COX3, ND4/4L, CytB) or ssDNA probes (ssCytB) of *DmMTPAP* KO larvae (*DmMTPAP*^KO^) and control (wt and FM7,Tb) larvae at 4 days ael. (B) Quantification of mitochondrial tRNA steady-state levels in Northern Blot experiments in control (daGAL4 control, RNAi #1 control) and *DmMTPAP* KD (*DmMTPAP* RNAi #1) 5-day-old larvae. (C) *De novo* mitochondrial transcription in isolated mitochondria of control and *DmMTPAP* KD larvae at 5 days ael. Western blotting of VDAC in the input samples was used as a loading control. (D) qPCR of mtDNA steady-state levels in *DmMTPAP* KD (*DmMTPAP* RNAi #1, *DmMTPAP* RNAi #2) and control (daGAL4 control, RNAi #1 control, RNAi #2 control) larvae at 5 days ael.
(TIF)

S4 Fig *De novo* mitochondrial translation in DmMTPAP KD larvae. In organello labelling of mitochondrial translation products on isolated mitochondria from (A) DmMTPAP RNAi #1 and (B) *DmMTPAP* KD RNAi #2 larvae and their corresponding controls at 5 days ael. Labelling was performed for 60 min (pulse), followed by a 3-hour chase with cold methionine. Coomassie staining was performed to ensure equal loading of the gels.
(TIF)

S5 Fig Analysis of the OXPHOS complex assembly in DmMTPAP KD larvae. (A) BN-PAGE and in gel staining of Complex I and Complex IV activities in mitochondrial protein extracts from control (daGAL4 control, RNAi #1 control) and *DmMTPAP* KD (*DmMTPAP* RNAi #1) 5-day-old larvae. Coomassie staining of the gel was performed to ensure equal loading of the gels. (B) BN-PAGE and in gel staining of Complex I and Complex IV activities in mitochondrial protein extracts from control (daGAL4 control, RNAi #2 control) and *DmMTPAP* KD (*DmMTPAP* RNAi #2) 5-day-old larvae. Coomassie staining of the gel was performed to ensure equal loading of the gels (C) Complex V assembly was assessed in *DmMTPAP* KD (*DmMTPAP* RNAi #2) 5-day-old larvae by BN-PAGE, followed by Western blot analysis against the F1 subunit of Complex V. Coomassie staining was used to ensure equal loading of the gels.
(TIF)
