# Supplementary material for: Mitochondrial Polyadenylation Is a One-Step Process Required for mRNA Integrity and tRNA Maturation
Source: PLoS Genet. 2016 May 13;12(5):e1006028. doi: 10.1371/journal.pgen.1006028 (PMC4866704; doi:10.1371/journal.pgen.1006028)
Supplement: S2 Table — (DOCX) [file pgen.1006028.s002.docx]

| **tRNA** | **3’ end position** | **3’ end addition** | **Number of clones** |
| --- | --- | --- | --- |
| **wt** | | | |
| **tRNA^Val^** | 0 | CCA | 21 |
|  | 0 | CC | 1 |
|  | 0 | C | 1 |
|  | 0 | CCACCTTTCC | 1 |
|  | 0 | CCAA | 1 |
|  | -3 | - | 2 |
|  | -3 | CAATTT | 1 |
|  | -3 | CT | 1 |
|  | -5 | - | 1 |
|  | -12 | - | 1 |
| **tRNA^Cys^** | 0 | CCA | 32 |
|  | 0 | CC | 8 |
|  | 0 | - | 1 |
|  | 0 | CT | 1 |
|  | 0 | CCATG | 1 |
|  | 0 | CCT | 1 |
|  | 0 | CT | 1 |
| ***DmMTPAP*^KO^** | | | |
| **tRNA^Val^** | 0 | CCA | 14 |
|  | 0 | CC | 4 |
|  | 0 | C | 2 |
|  | 0 | CCACAA | 1 |
|  | 0 | CCACATCTC | 1 |
|  | 0 | CCAATTAAAAGGTA | 1 |
| **tRNA^Cys^** | 0 | CCA | 6 |
|  | 0 | CC | 15 |
|  | 0 | CCT | 3 |
|  | 0 | CT | 2 |
|  | -3 | - | 1 |

**S2 Table. tRNA 3’RACE in wt and *DmMTPAP*^KO^ larvae**
