# Supplementary material for: Mitochondrial Polyadenylation Is a One-Step Process Required for mRNA Integrity and tRNA Maturation
Source: PLoS Genet. 2016 May 13;12(5):e1006028. doi: 10.1371/journal.pgen.1006028 (PMC4866704; doi:10.1371/journal.pgen.1006028)
Supplement: S3 Table — (DOCX) [file pgen.1006028.s003.docx]

**Table S3. Taqman Probes and oligonucleotides used in this study**

| **Taqman Assays** | | |  |  |
| --- | --- | --- | --- | --- |
| DmMTPAP | Dm01829473m1 | |  |  |
| DmRpL32 | Dm02151827_g1 | |  |  |
| **Primers for cloning of the homology arms in pGX-attP** | | |  |  |
| DmMTPAP_5’arm_F | TATCTCGCACGAAGCCCAAGTTCTCGTAGAGCCGCAAAGCCGGCTGGTTA**GCATGC**CACAACATACGAGCCGGAAGCATA | |  |  |
| DmMTPAP_5’arm_R | AGGATGTAGTGCAGGGTCTCGTCCTGCCGCACGCAGTAGTGGTGGGCACC**GCGGCCGC**ATGTGCGCGGAACCCCTATTTG | |  |  |
| DmMTPAP_3’arm_F | ATTGACTAATGTAACCGATCCAGGAGTGTTAGGAAGATGACTAACTAACG**AGATCT**CACAACATACGAGCCGGAAGCATA | |  |  |
| DmMTPAP_3’arm_R | CACTAGTCCAGCCATTGATTCGCCAAATCCCATTACGTTAAGGGCGCATC**CCTAGG**ATGTGCGCGGAACCCCTATTTG | |  |  |
| **Primers for PCR screening and Southern Blot probe to map *DmMTPAP*^KO^ lines** | | |  |  |
| SouthernProbe_F | AGTAGAGCAAACCCAAGGCG | |  |  |
| SouthernProbe_R | GCAGAGATTTGGACTTGGCG | |  |  |
| MTPAP_ScrPCR1_F | GCCAACAGACAGACGAATGG | |  |  |
| MTPAP_ScrPCR1_R | ACTCTCCCACGATGTAGTGC | |  |  |
| MTPAP_ScrPCR2_F | GGATGAAATTGCCATCAAAGCTC | |  |  |
| MTPAP_ScrPCR2_R | ACTGGGCTCGACTTAAAGGC | |  |  |
| MTPAP_ScrPCR3_F | CTGGCTCTAAGACTTCGGGC | |  |  |
| MTPAP_ScrPCR3_R | CTTTCTCATAGCCCACACGCT | |  |  |
| **Primers for qRT-PCR quantification of mRNAs** | | |  |  |
| DmMTPAP_F | CATCAAGTACCACCATGAGCA | |  |  |
| DmMTPAP_R | TCCGACATATAAAAGCCAGTGAG | |  |  |
| His2B_F | CTGGCAAGGCTCAGAAGAAC | |  |  |
| His2B_R | AAATTCCGGTGTCAGGATGG | |  |  |
| Tsp2A_F | GGCCATGGGATTATCTCGAC | |  |  |
| Tsp2A_R | TTTTGCCCTCGAAGAACCAG | |  |  |
| CG12773_F | CGGATCGATATGCTAAGCTGT | |  |  |
| CG12773_R | TACTCGTTCATCCGTTTGCC | |  |  |
| CG11412_F | GGTCACCAAGGCCATAGAGG | |  |  |
| CG11412_R | TTATCTCGCACGAAGCCCAA | |  |  |
| **Primers for qPCR quantification of mtDNA** | | |  |  |
| 12S_F | GATAACGACGGTATATAAACTGATTACA | |  |  |
| 12S_R | GAGGAACCTGTTTTTTAATCGA | |  |  |
| COX3_F | CAGACTCAATTTATGGATCAACATT | |  |  |
| COX3_R | AAAGTTGTTCCGATTAATACATGAA | |  |  |
| RP49_F | CGGATCGATATGCTAAGCTGT | |  |  |
| RP49_R | CGACGCACTCTGTTGTCG | |  |  |
| **Primers for 3’RACE** | | |  |  |
| Linker | ATGTGAGATCATGCACAGTCATA | |  |  |
| Anti-linker | GACTGTGCATGATCTCAC | |  |  |
| COX1_F | GCTTACACAACATGAAATATTGTATCA | |  |  |
| CytB_F | CAAATTTATTGGGAGACCCTGA | |  |  |
| tRNA-Val_F | ACATTGAAAAGATTTTTGTGCAA | |  |  |
| tRNA-Cys_F | TCAAACTGCAATTTTGAAGGAGT | |  |  |
| **Primers for circularization and RT-PCR of mt-RNAs** | | |  |  |
| ATP6/8_R1 | ATAAATAATAATAATCATCTAATA | Reference 23 |  |  |
| ATP6/8_F | AATCTTATGTGTTTGCTGTAT | Reference 23 |  |  |
| ATP6/8_R2 | TTTAATTCATTAGATTTAGGTG | Reference 9 |  |  |
| ND6_R | AATTTTTTTAGGAGGAATACTTG |  |  |  |
| ND5_R1 | AATGAATCGATTAATATGATTGTCATTT |  |  |  |
| ND5_F1 | TGGTCAATTAGTTGTAAAAAGTTTTGA | |  |  |
| ND5_R2 | AAAGTTGAATTATACTCCGTGG Reference 9 | |  |  |
| ND5_F2 | GAAACAAGTCCTAAACCATCTCA Reference 9 | |  |  |
| ND4/4L_R | CCTTCACATACTCTAAATGTCAAAAAT | |  |  |
| ND4/4L_F | TGGTCAATTAGTTGTAAAAAGTTTTGA |  |  |  |
| ND1_R | AGATAATAAAGGATAAGTT | Reference 23 |  |  |
| ND1_F1 | GGAACTTTACCTCGATTT | Reference 23 |  |  |
| ND1_F2 | AAGGATCCGATTAGTTTCAGCT | Reference 9 |  |  |
| 16S_R1 | TAATAAACACTGATACACAAGGTACAA | Reference 23 |  |  |
| 16S_F1 | GACCTCGATGTTGGATTAAGATATAAT | Reference 23 |  |  |
| 16S_R2 | ACATGATCTGAGTTCAAACCGG | Reference 9 |  |  |
| 16S_F2 | TTTAATAAACACTGATACACAAG | Reference 9 |  |  |
| 12S_R | TAACCGCGACTGCTGGCAC | Reference 9 |  |  |
| 12S_F | TTTAATCGATAATCCACGATGG | Reference 9 |  |  |
