# Supplementary figures and images for: Mitochondrial Polyadenylation Is a One-Step Process Required for mRNA Integrity and tRNA Maturation
Source: PLoS Genet. 2016 May 13;12(5):e1006028. doi: 10.1371/journal.pgen.1006028 (PMC4866704; doi:10.1371/journal.pgen.1006028)

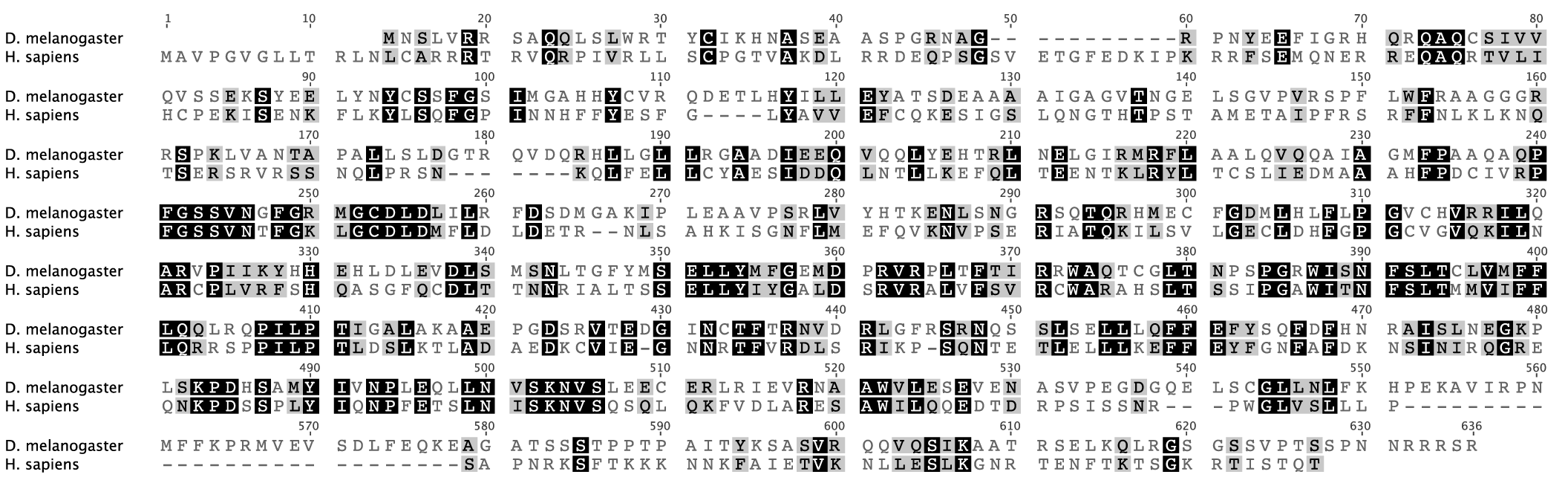

Supplement: S1 Fig — Clustal W alignment of human mitochondrial poly(A) polymerase (bottom, AAH61703) and its D. melanogaster ortholog (top, AAF45607). (TIF) [file pgen.1006028.s004.tif]

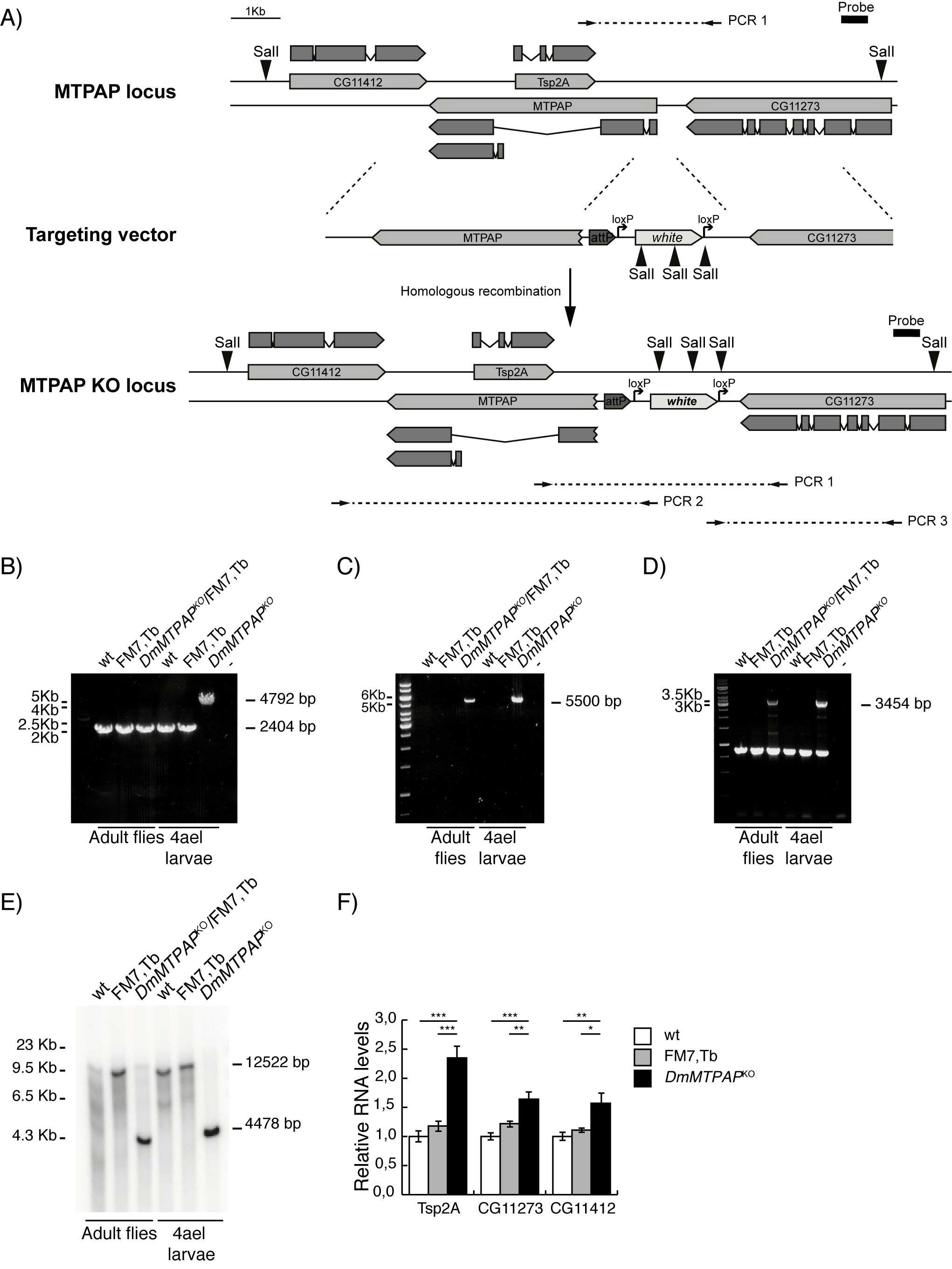

Supplement: S2 Fig — (A) Schematic diagram of the MTPAP locus, the targeting vector for homologous recombination and the KO locus. (B-D) Homologous targeting confirmation by PCR on control (wt and FM7,Tb), heterozygous (DmMTPAPKO/FM7,Tb) and hemizygous knockout (DmMTPAPKO) samples. Primers (see M&Ms) were chosen to either span the deleted region (B and PCR1 in S1A) or the homologous arms of the targeting vector (C, D and PCR 2,3 in S1A). (E) Southern blot of SalI digested genomic DNA probed as shown in S1A. (F) qRT-PCR of expression levels of genes adjacent to the deleted MTPAP locus. (TIF) [file pgen.1006028.s005.tif]

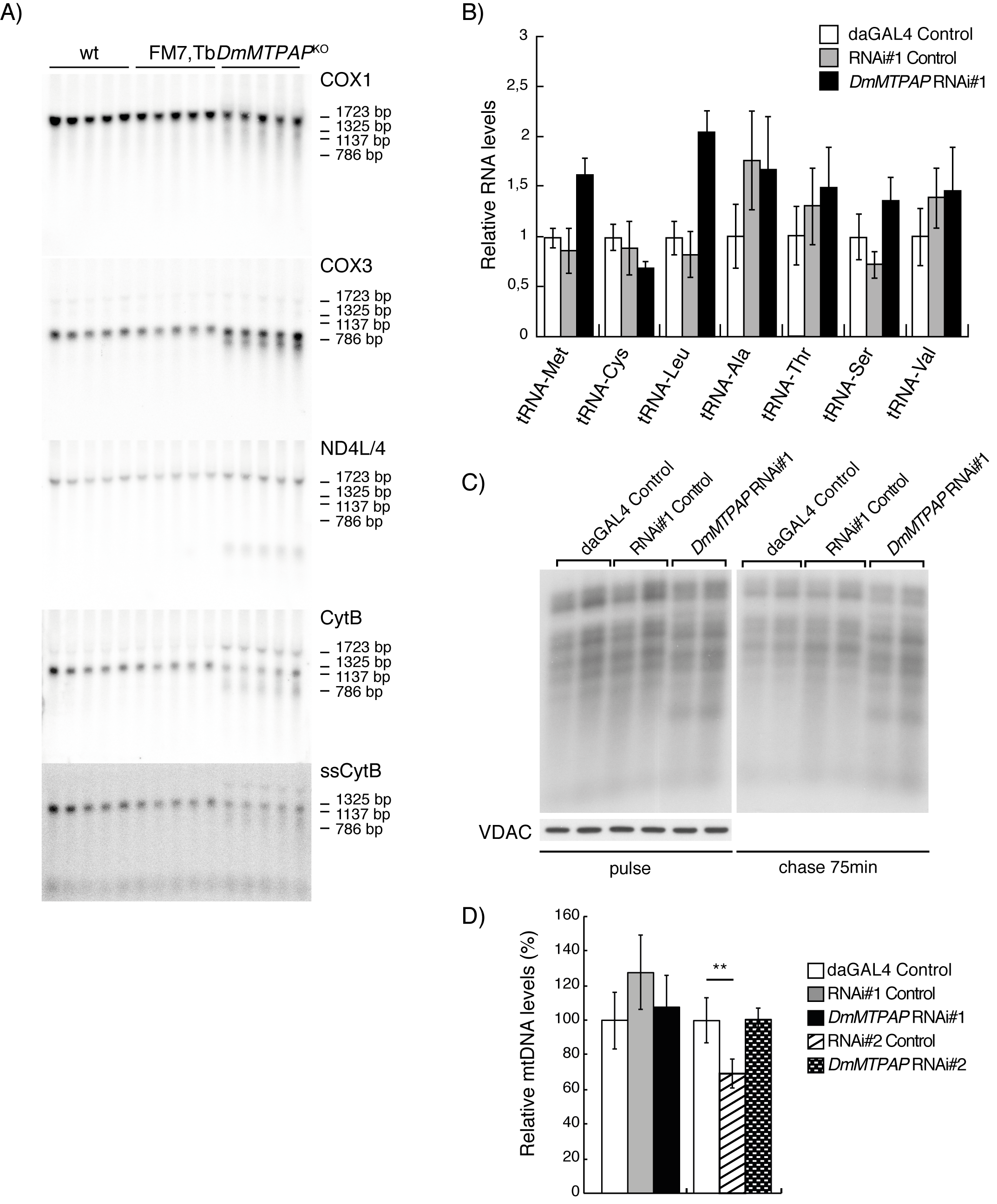

Supplement: S3 Fig — (A) Northern Blot analysis of mitochondrial mRNAs using dsDNA probes (COX1, COX3, ND4/4L, CytB) or ssDNA probes (ssCytB) of DmMTPAP KO larvae (DmMTPAPKO) and control (wt and FM7,Tb) larvae at 4 days ael. (B) Quantification of mitochondrial tRNA steady-state levels in Northern Blot experiments in control (daGAL4 control, RNAi #1 control) and DmMTPAP KD (DmMTPAP RNAi #1) 5-day-old larvae. (C) De novo mitochondrial transcription in isolated mitochondria of control and DmMTPAP KD larvae at 5 days ael. Western blotting of VDAC in the input samples was used as a loading control. (D) qPCR of mtDNA steady-state levels in DmMTPAP KD (DmMTPAP RNAi #1, DmMTPAP RNAi #2) and control (daGAL4 control, RNAi #1 control, RNAi #2 control) larvae at 5 days ael. (TIF) [file pgen.1006028.s006.tif]

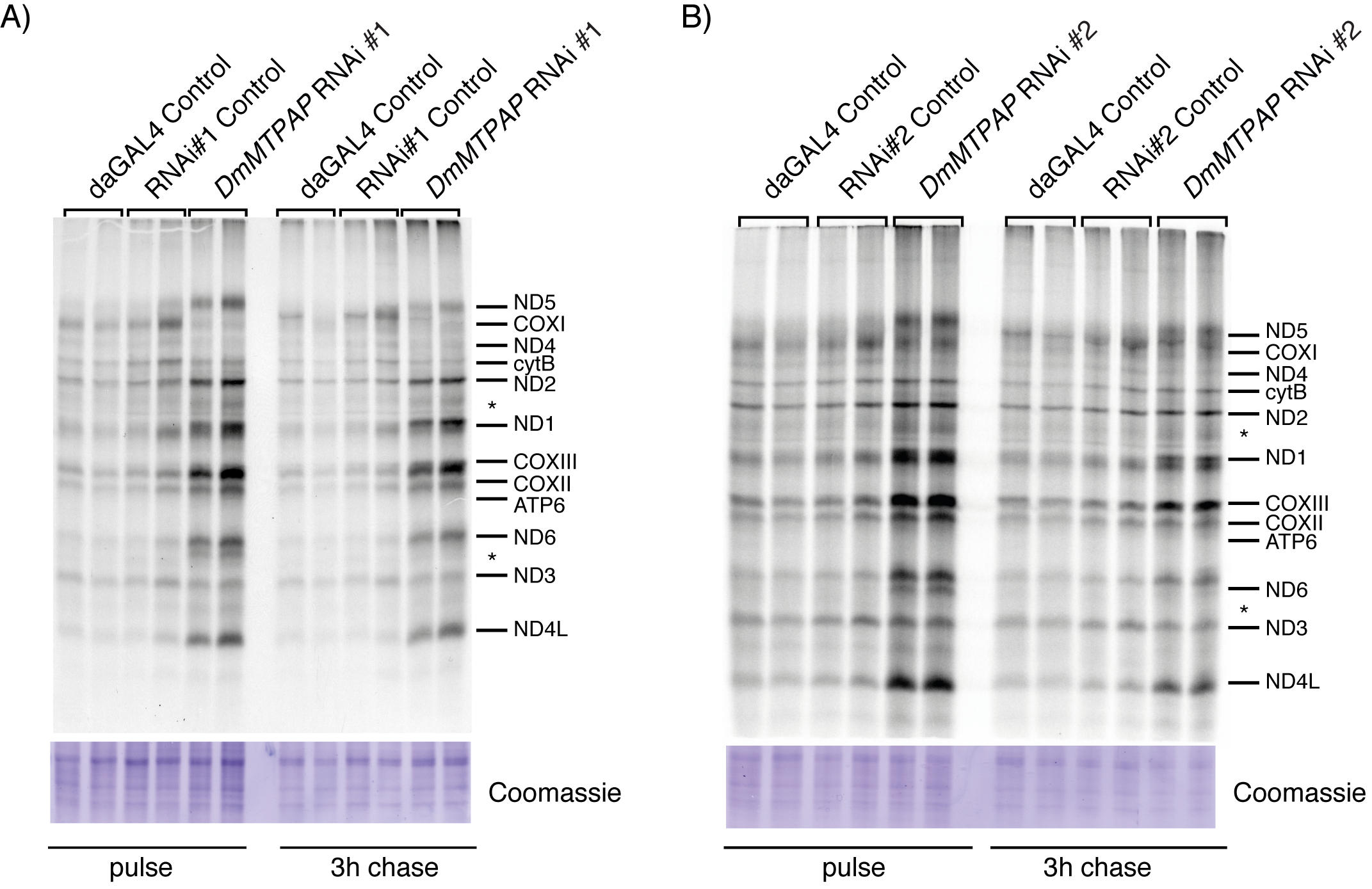

Supplement: S4 Fig — In organello labelling of mitochondrial translation products on isolated mitochondria from (A) DmMTPAP RNAi #1 and (B) DmMTPAP KD RNAi #2 larvae and their corresponding controls at 5 days ael. Labelling was performed for 60 min (pulse), followed by a 3-hour chase with cold methionine. Coomassie staining was performed to ensure equal loading of the gels. (TIF) [file pgen.1006028.s007.tif]

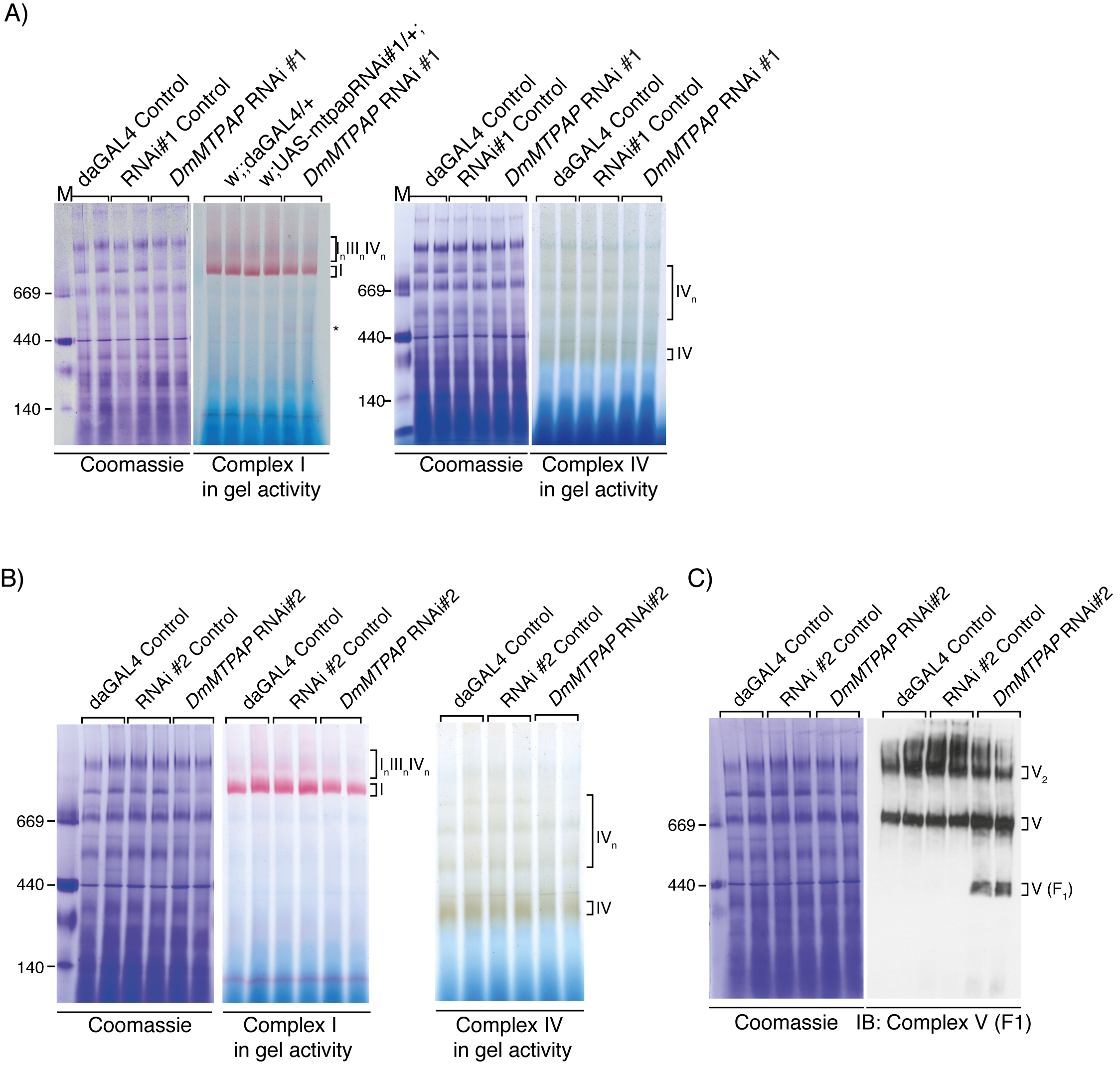

Supplement: S5 Fig — (A) BN-PAGE and in gel staining of Complex I and Complex IV activities in mitochondrial protein extracts from control (daGAL4 control, RNAi #1 control) and DmMTPAP KD (DmMTPAP RNAi #1) 5-day-old larvae. Coomassie staining of the gel was performed to ensure equal loading of the gels. (B) BN-PAGE and in gel staining of Complex I and Complex IV activities in mitochondrial protein extracts from control (daGAL4 control, RNAi #2 control) and DmMTPAP KD (DmMTPAP RNAi #2) 5-day-old larvae. Coomassie staining of the gel was performed to ensure equal loading of the gels (C) Complex V assembly was assessed in DmMTPAP KD (DmMTPAP RNAi #2) 5-day-old larvae by BN-PAGE, followed by Western blot analysis against the F1 subunit of Complex V. Coomassie staining was used to ensure equal loading of the gels. (TIF) [file pgen.1006028.s008.tif]
